# Depressive symptoms among healthcare undergraduate students*

**DOI:** 10.1590/1518-8345.3210.3239

**Published:** 2020-02-14

**Authors:** Julia Zancan Bresolin, Graziele de Lima Dalmolin, Silvio José Lemos Vasconcellos, Edison Luiz Devos Barlem, Rafaela Andolhe, Tania Solange Bosi de Souza Magnago

**Affiliations:** 1 Prefeitura Municipal de Santa Maria, Secretaria Municipal de Saúde, Santa Maria, RS, Brazil.; 2 Universidade Federal de Santa Maria, Departamento de Enfermagem, Santa Maria, RS, Brazil.; 3 Universidade Federal de Santa Maria, Departamento de Psicologia, Santa Maria, RS, Brazil.; 4 Universidade Federal do Rio Grande, Escola de Enfermagem, Rio Grande, RS, Brazil.

**Keywords:** Depression, Students, Students, Health Occupations, University, Education Higher, Stress, Psychological, Depressão, Estudantes, Estudantes de Ciências da Saúde, Universidade, Educação Superior, Estresse Psicológico, Depresión, Estudiantes, Estudiantes del Área de la Salud, Universidade, Educación Superior, Estrés Psicológico

## Abstract

**Objective:**

to identify the intensity of depressive symptoms and their associated factors in healthcare undergraduate students.

**Method:**

cross-sectional study developed with undergraduate health students from a public higher education institution using the Beck Depression Inventory-version II and a student characterization questionnaire. The study involved 792 participants. For data analysis, we used descriptive statistics, chi-squared test and Poisson regression.

**Results:**

the intensity of depressive symptoms was moderate to severe in 23.6% of the students, associated with the non-performance of physical and leisure activities and with speech therapy and nursing courses.

**Conclusion:**

several factors may be associated with depression, thus, further investigation into the related factors that cause its emergence in this period of life is necessary, as well as raising institutional awareness and developing strategies at the personal and group level to promote well-being, improve time management and interpersonal relationships, in order to achieve better academic results and personal development.

## Abstract (pt)

**Objetivo:**

identificar a intensidade de sintomas depressivos e seus fatores associados em estudantes universitários da área da saúde.

**Método:**

estudo transversal desenvolvido com estudantes dos cursos de graduação da área da saúde de uma instituição de ensino superior pública por meio do Inventário de Depressão de Beck - versão II e um questionário de caracterização dos estudantes. Participaram do estudo 792 estudantes. Utilizaram-se, para a análise dos dados, estatística descritiva, teste do qui-quadrado e regressão de Poisson.

**Resultados:**

a intensidade dos sintomas depressivos mostrou-se entre moderada e grave em 23,6% dos estudantes, associada à não realização de atividades física e de lazer e aos cursos de fonoaudiologia e enfermagem.

**Conclusão:**

diversos fatores podem estar associados à depressão, assim, tornam-se necessárias maiores investigações quanto aos fatores relacionados que provocam seu surgimento nesse período de vida, bem como estar atento institucionalmente e desenvolver estratégias em nível pessoal e grupal que promovam bem-estar, melhoria da gestão do tempo e relações interpessoais, acarretando melhor aproveitamento e desenvolvimento do estudante.

## Abstract (es)

**Objetivo:**

identificar la intensidad de síntomas depresivos y sus factores asociados en estudiantes universitarios del área de la salud.

**Método:**

estudio transversal desarrollado con estudiantes de los cursos de grado del área de la salud de una institución de enseñanza superior pública por medio del Inventario de Depresión de Beck-versión II y un cuestionario de caracterización de los estudiantes. Participaron en el estudio 792 estudiantes. Para el análisis de los datos, se utilizaron estadística descriptiva, prueba chi cuadrado y regresión de Poisson.

**Resultados:**

la intensidad de los síntomas depresivos se mostró entre moderada y grave en el 23,6 % de los estudiantes, asociada a la no realización de actividades físicas y de ocio y a los cursos de fonoaudiología y enfermería.

**Conclusión:**

diversos factores pueden estar asociados a la depresión, así, se requieren más investigaciones respecto a los factores relacionados que provocan su surgimiento en este período de vida, así como estar atento institucionalmente y desarrollar estrategias en nivel personal y grupal que promuevan el bienestar, la mejora de la gestión del tiempo y las relaciones interpersonales, lo que resulta en un mejor aprovechamiento y desarrollo del estudiante.

## Introduction

Depression is acknowledged as a public health issue that can compromise an individual’s daily activities, especially regarding social life^(^ 1 ^)^. According to the Diagnostic and Statistical Manual of Mental Disorders^(^ 2 ^)^, depression is characterized by loss of interest and pleasure in performing almost all activities; depressed mood; changes in appetite and sleep; decreased energy; feelings of worthlessness and guilt; difficulty in reasoning, concentration and decision making. Depression can also be understood, according to the cognitive model, by three fundamental aspects, namely the cognitive triad, dysfunctional cognitive self-schema and cognitive distortions, which refer to a negative view of oneself, life and the future^(^ 3 ^)^.

Depression, as well as other forms of illness, has a high chance of manifesting itself in early adulthood, and sometimes can occur concurrently with undergraduate studies. This is due to the fact that young people undergo major changes and losses, expected at this stage of a person’s development, among them the withdrawal from the family and social circle and the adaptation to the new routine, which can trigger a crisis situation^(^ 4 ^)^.

The new situations experienced by students during their academic daily life that require adjustment include the demands of the course itself and/or of the institution due to the high number of subjects to be studied and the competition of the labor market. All of these factors, in addition to family demands, add to the fear of professional failure and loss of prestige, which may trigger depressive symptoms^(^ 5 ^)^.

Healthcare undergraduate students, in particular, face still other aggravating factors, such as initiation to clinical practice, contact with patients with serious and terminal diseases, ethical conflicts, social vulnerabilities and health system weaknesses. These factors tend to contribute to the onset of interpersonal difficulties and of symptoms of psychological distress, such as depression^(^ 6 ^)^.

Given this context, a high prevalence of depression has been observed, such as shown in a systematic review involving 167 cross-sectional and 16 longitudinal studies from 43 countries, involving over 100,000 students, which identified a 27.2% average prevalence of depression, ranging from 9.3% to 55.9%^(^ 7 ^)^. Additionally, 36% of the students finishing the first year of a Dentistry undergraduate course and 45.7% of the students of a medical course were identified with at least some degree of depression^(^ 8 ^-^ 9 ^)^.

Among the related factors involved were the desire to switch undergraduate courses, difficulties in social relationships and evaluating the teaching environment as an environment with many problems^(^ 8 ^)^. The study also pointed other predictors of depressive symptoms in college students: sex, basal depression, neuroticism or psychoticism, automatic negative thoughts or obsessive negative rumination, dysfunctional attitude, childhood abuse, sexual abuse and stressful life events^(^ 10 ^)^.

Given the data presented, it is important to consider the onset of depressive symptoms in higher education students in order to find coping strategies for reducing the suffering and anguish that this period may cause, as well as for preventing these future professionals from entering working life already sick.

Importantly, there is a gap in knowledge on depression in healthcare undergraduate students. We searched the Virtual Health Library (VHL), using the descriptors “*estudantes de ciências da saúde*” (health sciences students) and “*depressão*” (depression), between 2012 and 2016. We initially found 16 articles, but two of them were duplicates, leaving 15 articles. Of these, 10 were indexed in the Medical Literature Analysis and Retrieval System Online (MEDLINE), three in the Latin American and Caribbean Health Sciences Literature (LILACS) and two in the *Índice Bibliográfico Español en Ciencias de la Salud*(IBECS - Spanish Bibliographic Index on Health Sciences). Of these studies, six did not involve healthcare students^(^ 11 ^-^ 16 ^)^, six had another topic as their study focus^(^ 17 ^-^ 22 ^)^ and two analyzed only one healthcare course^(^ 23 ^-^ 24 ^)^. Only one study evaluated four healthcare courses, but was conducted in Saudi Arabia^(^ 25 ^)^. Thus, we found no Brazilian study evaluating depressive symptoms in undergraduate students in all healthcare courses.

The present study, therefore, was guided by the research question: What is the intensity of depressive symptoms and their associated factors in healthcare undergraduate students? Our objective in this study was to identify the intensity of depressive symptoms and their associated factors in healthcare undergraduate students.

## Method

This is a cross-sectional study, conducted at a public university in the interior of the state of Rio Grande do Sul, Brazil. The university offers basic, technical and technological education, undergraduate and postgraduate courses, and has 28,908 enrolled students.

The study was conducted with healthcare students, encompassing undergraduate courses in Nursing, Pharmacy, Physiotherapy, Speech Therapy, Medicine, Dentistry and Occupational Therapy. The population totaled 2,334 students.

Inclusion criteria were being enrolled and regularly attending the course and being 18 or older. Being off course for any reason during data collection was an exclusion criterion.

To minimize possible sampling biases, we performed a finite population sampling calculation to estimate the minimum number of participants required for the study. We considered a 95% confidence level; sampling error=0.5 points; standard deviation=7.32^(^ 26 ^)^; and total student population=2334. Thus, we estimated a sample (n) of 609 participants. Considering possible losses, this number was increased by 20%, which resulted in 731 students. For selecting the students, we opted for a probabilistic cluster sampling, since the units of analysis were in the same physical location, that is, we selected students at the beginning, middle and end of each undergraduate course.

Data was collected from April to July 2017, after institutional authorization and approval by the local Research Ethics Committee. Data collection was conducted developed in classrooms by the authors and other previously trained members of the research group, with the authorization and prior appointment with the teachers. The authors were pursuing a graduate degree in nursing and the research collaborators were undergraduate healthcare students at ​​the same institution. The research instrument was a form sent by e-mail to students of the last semesters of undergraduate courses. During this period, the students were not attending classes in the teaching units, as they were interning in health services units and developing their end of course papers. To ensure students’ greater adherence, it was requested that the coordinators of each course send e-mail inviting them to participate in the study and providing the web address for the form to be completed.

The instrument used was the self-report Beck Depression Inventory version II (BDI II), consisting of a set of 21 statements about common symptoms of depression, in which the participant should identify depressive symptoms experienced in the last 15 days, including the day of the inventory application^(^ 27 ^-^ 28 ^)^.

Depression was measured by summing the scores of the individual items on the scale, which provided a measure of the intensity of depressive symptoms. Symptoms were classified according to severity levels: from zero to 13 points, minimum/no depression; 14 to 19 points, mild depression; from 20 to 28 points, moderate depression; and finally, from 29 to 63 points, severe depression^(^ 29 ^)^. In addition, the total average score of the instrument was calculated.

We also used a questionnaire to characterize the students’ profile through sociodemographic variables (gender, age, race, marital status and city of origin), academic variables (course, semester, scientific initiation workload, whether they receive a scholarship or do an unpaid voluntary internship) and health habits (physical and leisure activities, medication, clinical condition diagnosed by doctor, body mass index, means of transport to the university, time spent using cell phone and computer).

To analyze the data we used Excel^®^, with independent double typing. Subsequently, for data analysis, we used the PASW Statistic^®^ program (Predictive Analytics Software, from SPSS Inc., Chicago, USA) version 18.0 for Windows. For analyzing qualitative variables, relative and absolute frequencies were used, and for quantitative variables, measures of position and dispersion, according to the normality of the data.

To associate sociodemographic, academic and health habits variables with depressive symptom levels, the Chi-squared test was used, and associations with p<0.05 were considered statistically significant. For multivariate analysis, we used Poisson regression (stepwise method), estimating the gross and adjusted prevalence ratios (RPb and RPaj) and the respective confidence intervals (95%CI). The variables with p<0.20 were included in the gross regression, and in the adjusted regression those with p<0.15, also adopting those with p <0.05 as statistically significant. In these two analyzes, depressive symptoms were grouped into two levels of intensity, minimum/mild (0-19) and moderate/severe (20-63).

The research project was approved by the Research Ethics Committee under Certificate of Presentation for Ethical Appreciation (CAAE) n^o^. 63473317.1.0000.5346 and Approval Opinion No. 1,888,749 of 01/11/2017. All ethical precepts of research involving human beings were respected according to Resolution 466/12, ensuring the voluntariness of participation, anonymity of participants and the confidentiality of the data obtained.

## Results

The study involved 792 participants (n = 744, 93.9%; and via e-mail, n = 48, 6.1%). Most were female (n = 591, 74.6%), aged between 18 and 22 years (n = 560, 70.7%), white (n = 632 -79.8%), single (n = 749, 94.6%), without children (n = 767, 96.8%), coming from cities of Rio Grande do Sul, Brazil (n = 692, 87.3%).

From the Nursing course, 117 (14.8%) students participated; Pharmacy, 105 (13.3%); Physiotherapy, 70 (8.8%); Speech Therapy, 63 (8.0%); Medicine, 192 (24.2%); Dentistry, 143 (18.0%); and Occupational Therapy, 102 (12.9%).

Depression was moderate to severe in 187 (23.6%) of healthcare undergraduate students. The course with the highest rate of moderate to severe depressive symptoms was Speech Therapy (n = 30, 47.6%), followed by Nursing (n = 40, 34.2%), Pharmacy (n = 24, 22.9 %), Medicine (n = 40, 20.9%), Occupational Therapy (n = 21, 20.5%), Physiotherapy (n = 11, 15.8%) and Dentistry (n = 21, 14.7%). Table 1 shows the classification of depressive symptoms according to each course.

**Table 1:** Classification of depressive symptoms according to each health course. Santa Maria, RS, Brazil, 2017

Course	No Depression n (%)	Mild Depression n (%)	Moderate Depression n (%)	Severe Depression n (%)
Nursing	53 (45.3)	24 (20.5)	31 (26.5)	9 (7.7)
Pharmacy	63 (60)	18 (17.1)	15 (14.3)	9 (8.6)
Physiotherapy	46 (65.7)	13 (18.6)	9 (12.8)	2 (2.9)
Speech Therapy	16 (25.4)	17 (27.0)	18 (28.6)	12 (19.0)
Medicine	118 (61.5)	34 (17.7)	23 (12.0)	17 (8.8)
Dentistry	99 (69.2)	23 (16.1)	19 (13.3)	2 (1.4)
Occupational Therapy	51 (50.0)	30 (29.5)	13 (12.7)	8 (7.8)

Regarding the individual items of the scale, we describe those with the highest prevalence, showing which are the symptoms that most stood out among the students. We found that 35 (4.4%) of the students blamed themselves for everything bad that happened; 93 (11.7%) had difficulty making any decision; 38 (4.8%) had changes in their sleep pattern, that is, they slept most of the day or woke up an hour or two earlier and could not sleep again. Regarding irritability, 44 (5.6%) said they were angry all the time, 49 (6.2%) had no appetite or wanted to eat all the time and 55 (6.9%) felt too tired to do most of the things they used to do.

Table 2 presents the associations of sociodemographic, health and academic variables with depressive symptoms, showing that the highest intensity of depression affected females; obese students; those with a medical condition diagnosed by a doctor; those who used the bus as a means of transportation; those who did not perform physical activities; those who did not have leisure activities; and among Speech Therapy and Nursing students.

**Table 2:** Associations of sociodemographic, health and academic variables with depressive symptoms in healthcare undergraduate students. Santa Maria, RS, Brazil, 2017

Variables	Depression - n (%)		p*
	Minimum/Mild	Moderate/Severe	
Gender			
Female	438 (74.1)	153 (25.9)	0.009
Male	167 (83.1)	34 (16.9)	
Clinical condition diagnosed by doctor			
Yes	160 (69.9)	69 (30.1)	0.007
No	445 (79.0)	118 (21.0)	
Uses medication			
Yes	228 (71.7)	90 (28.3)	0.01
No	377 (79.5)	97 (20.5)	
Transport			
Bus	300 (72.6)	113 (27.4)	0.01
Other	305 (80.5)	74 (19.5)	
Physical activities			
Yes	240 (84.5)	44 (15.5)	<0.001
Occasionally	203 (79.9)	51 (20.1)	
No	162 (63.8)	92 (36.2)	
Leisure			
Yes	250 (87.4)	36 (12.6)	<0.001
Occasionally	318 (72.4)	121 (27.6)	
No	37 (55.2)	30 (44.8)	
Course			
Speech Therapy	33 (52.4)	30 (47.6)	
Nursing	77 (65.8)	40 (34.2)	
Pharmacy	81 (77.1)	24 (22.9)	
Medicine	152 (79.2)	40 (20.8)	
Occupational Therapy	81 (79.4)	21 (20.6)	
Physiotherapy	59 (84.3)	11 (15.7)	
Dentistry	122 (85.3)	21 (14.7)	
BMI^†^			
Low weight	33 (73.3)	12(26.7)	<0.001
Adequate weight	426 (80.2)	105(19.8)	
Overweight	111 (72.1)	43(27.9)	
Obesity	35 (57.4)	26(42.6)	
Cellphone time			
Up to 5 h	327 (78.2)	91 (21.8)	00.20
6 h or more	278 (74.4)	95 (25.6)	
Computer time			
Up to 2 h	315(78.6)	86 (21.4)	
3 h	285 (74.0)	100 (26.0)	40.15
Semester			
1st to 2nd sem.	222 (79.9)	56 (20.1)	
3rd to 6th sem.	240 (73.4)	87 (26.6)	0.17
7th to 12th sem.	143 (76.9)	43 (23.1)	

* p = Chi-squared test;

† BMI = Body mass index

Among other variables, we tested age, origin, marital status, number of children, alcohol and tobacco consumption, receiving a scholarship, number of shifts worked in the month, performing voluntary internship and number of hours of practical classes, which showed no significant difference, presenting p>0.20.

Subsequently, we performed a Poisson regression. The following are the variables that were included in the gross analysis and their respective results: gender (female: RPb=1.077; 95%CI: 1.022-1.135; p = 0.006), means of transport (bus: RPb=1.066; 95%CI: 1.016-1.117; p=0.009), medical diagnosis (yes: RPb=1.076; 95%CI: 1.020-1.135; p=0.007), medication (yes: RPb=1.065; 95%CI: 1.014-1.119; p=0.012 ), cellphone time (six hours or more: RPb=1.032; 95%CI: 0.983-1.082; p=0.206), computer time (three hours or more: RPb=1.037; 95%CI: 0.999-1.088; p=0.135 ), physical activity (no: RPb=1.179; 95%CI: 1.114-1.248; p=0.000; occasionally: RPb=1.040; 95%CI: 0.984-1.098; p=0.164).

Other results were: a) Leisure (no: RPb=1.286; 95%CI: 1.176-1.406; p=0.000; occasionally - RPb=1.133; 95%CI: 1.081-1.188; p=0.00), Body Mass Index (BMI) (obesity: RPb=1.191; 95%CI: 1.087-1.305; p=0.000; overweigh: RPb=1.068; 95%CI: 1.004-1.137; p=0.038; Low Weight: RPb=1.058; 95%CI: 0.951-1.166; p=0.300); b) Course (Speech Therapy: RPb=1.287; 95%CI: 1.167-1.419; p=0.000; Nursing: RPb=1.170; 95%CI: 1.078-1.270; p=0.000; Pharmacy: RPb=1.071; 95%CI: 1.986-1.164; p=0.103; Medicine: RPb=1.054; 95%CI: 0.983-1.129; p=0.140; Occupational Therapy: RPb=1.051; 95%CI: 1.968-1.142; p=0.233; Physical Therapy: RPb=1.009; 95%CI: 0.923-1.103, p=0.845); c) Semester (3rd to 6th: RPb=1.054; 95%CI: 0.998-1.113; p=0.060; 7th-12th: RPb=1.025; 95%CI: 1.962-1.091; p=0.446).

In the adjusted analysis, the variables were initially tested in their large subgroups, namely: 1) Sociodemographic (gender + transport); 2) Habits and health (medical diagnosis + medication + computer time + physical activity + leisure + BMI); and 3) Academic (course + semester). Those variables which remained associated with symptoms of depression with p<0.15, were submitted to a second adjusted analysis, including: gender (p=0.020), transport (p=0.027), medical diagnosis (p=0.127), medication (p=0.099); physical activity (p<0.001), leisure (no and occasionally: p<0.001), BMI (obesity p<0.001; overweight p=0.026); and course (speech therapy and nursing p<0.001, pharmacy p=0.125, medicine p=0.119).

In this second adjusted analysis, the variables of the different groups, sociodemographic, habits and health, and academics were included. After a gradual process of inclusion and exclusion, Table 3 shows those that remained associated with depressive symptoms, with p<0.05.

**Table 3:** Adjusted association according to sociodemographic, academic and health habits variables of healthcare undergraduate students. Santa Maria, RS, Brazil, 2017

Variable	Rpa J2*	95%CI^†^	p^‡^
Physical Activities			
No	1.128	1.062-1.197	0.000
Occasionally	1.007	0.954-1.064	0.971
Yes	1.000	---	---
Leisure			
No	1.209	1.103-1.325	0.000
Occasionally	1.097	1.046-1.151	0.000
Yes	1.000	---	---
Course			
Speech Therapy	1.146	1.029-1.277	0.013
Nursing	1.108	1.022-1.201	0.013
Pharmacy	1.026	0.947-1.111	0.531
Medicine	1.021	0.953-1.094	0.552
Occupational Therapy	0.990	0.910-1.077	0.808
Physiotherapy	0.544	0.974-0.894	1.061
Dentistry	1.00	---	---

* RPaj = Adjusted prevalence ratio;

† CI = Confidence interval);

‡ p = Chi-squared test

The set of adjusted analyzes, initially of the sociodemographic variables, showed higher prevalence rates for depression among female students and those who used bus as transportation. Regarding habits and health variables, the highest prevalence was among students who did not perform physical activity, who had no time for leisure and the obese. Regarding the academic variables, we identified that the students of Speech Therapy and Nursing showed a higher prevalence. Finally, after a second adjusted analysis, grouping variables of the three dimensions - sociodemographic, academic and health habits -, we found that students that did not perform physical activities did not have time for leisure, and that those who attended speech therapy and nursing showed a higher prevalence of depressive symptoms.

## Discussion

The overall prevalence of common symptoms of depression was moderate to severe in 23.6% of the students. A meta-analysis of 39 studies from 1997 to 2015, involving more than 32,000 Chinese university students, identified a global prevalence of depression of 23.8%, which was higher among medical students^(^ 30 ^)^. When considering health services, a systematic review of the national academic production highlighted the prevalence of depression in health workers between 15.4% and 40.5%, with higher scores among professionals working in higher risk areas and/or working in shifts^(^ 31 ^)^.

In this sense, we can observe that depression occurs in students and health workers. In this case, undergraduate students have presented difficulties of a different order, which may involve adaptation to the university context, distancing from family and friends, financial difficulties and identification with the course. In addition, in practical activities, students have greater daily proximity to diseases, death and terminality, experiencing socioeconomic difficulties of the population and the difficulties encountered in health services^(^ 32 ^)^.

These situations may trigger a more negative view of students’ adaptation to the university context, course and future profession, which may be related to the cognitive model of depression, in which the person shows pessimism about himself, the world and the future, has cognitive schema that distort reality, following unfavorable patterns, and cognitive errors that adapt reality to negative schema, which reinforce depressive beliefs^(^ 3 ^)^.

By entering this scheme, this negative and pessimistic view may increase throughout the undergraduate course if there is no appropriate intervention, as demands and responsibilities tend to increase, even in the final period of the course, with greater pressure and obligations, because after graduation, there is the challenge of entering the job market, studying for residency or public exams. Faced with these situations, the student may be in constant suffering and these elements may be related to depression. Symptoms such as pessimism, sadness, crying, self-criticism and irritability should also serve as a warning, but often go unnoticed in the academic environment^(^ 33 ^)^.

In this sense, it should be considered that depression is multifactorial, involving genetic as well as environmental factors in its pathophysiology. In some cases, genetic aspects seem to be predominant (presence of several affected members in the family); in others, environmental, social, economic and relational aspects seem to play a relevant role, as well as the ability to manage these factors^(^ 34 ^)^.

Among the aspects that may influence the development of common symptoms of depression in undergraduate students are social, economic, academic and health habits factors. In this sense, we identified associations of common symptoms of depression with female students, obese students, those who had a medical condition diagnosed by a doctor, those who used bus as a means of transport, those who did not perform physical activity, those who did not have leisure activities, and with the courses of Speech Therapy and Nursing. After performing a regression analysis grouping these variables, we identified students who did not perform physical activity, did not have time for leisure and attended speech therapy or nursing courses, whose prevalence rates of depressive symptoms were 12%, 20%, 14% and 10% higher, respectively.

Among medical students from the state of Ceará, those who performed physical activities sporadically or rarely were, respectively, 2.45 times and 3.04 times more likely to develop depression, compared with those who regularly engaged in physical activities. Enjoying leisure activities and having good social relationships with friends and family seems to be good for students’ mental health^(^ 35 ^)^. Thus, this shows that not having healthy lifestyle habits, with time for physical activities and leisure, is detrimental to students’ mental and physical health.

The course that presented the highest prevalence of depression was speech therapy, a result that may be associated with its high course load. Speech therapy students from Ceará also reported feelings of anxiety, frustration, insecurity, inability, anguish, sadness, fear and negative feelings, especially while initiating the course^(^ 36 ^)^. The Nursing course also presented a high prevalence of depression, which demonstrates that the course load, the student’s insertion in the hospital environment and the thoughts regarding the professional future can cause these students to develop psychic disorders^(^ 37 ^)^.

The required classroom hours and the academic activities, such as study routines and work assignments, which need to be done outside the classroom may overload the student^(^ 38 ^)^. Therefore, it is important to consider that physical inactivity, along with lifestyle and anxiety and depression levels are risk factors for the onset or aggravation of various diseases, especially chronic degenerative diseases^(^ 39 ^)^. Students with certain personality traits who cannot maintain a balanced routine of academic and personal activities remain more isolated, anxious and develop eating disorders leading to excessive weight gain or loss. Thus, the student’s mental health is compromised, which may cause the onset of disorders such as depression. Among US students, the variables that stood out for stress, anxiety and depression were the pressure for academic performance, professional success and plans to pursue graduate studies; and most of them were transfer students who lived outside of the university campus^(^ 40 ^)^.

As for the variables identified in association with depression, women showed a prevalence of moderate/severe depression of 25.9%, and in gross regression showed a prevalence 7% higher compared with males. This may be associated with the fact that women reach physical and emotional maturity earlier, thus facing the challenges with more responsibility and commitment, compared with males. Concerns begin to emerge in undergraduate studies, as it is a decisive period that will define both one’s professional and personal future^(^ 41 ^)^.

We also found that students who used bus as a means of transportation to the university showed a prevalence of moderate/severe depression of 27.4%; and compared with those using other means, they showed a 6% higher prevalence. This may be associated with the need to wake up earlier, commuting alone through empty streets, urban violence, overcrowding at peak times, longer commuting times, and more. Bus users also report dissatisfaction; discomfort and stress with the always-packed buses; intolerable waiting time; lack of commitment and education of bus drivers; and lack of a pleasant structure^(^ 42 ^-^ 43 ^)^.

The identification of variables associated with depression among university students allows the development of strategies to prevent and promote their psychic health. With this, the process of teaching/learning can be optimized.

As limitations of the study, we highlight the difficulty of access and low response rates of students who were in the internship period, because they were not attending classroom activities in the teaching unit, but were working in health service units, both in the municipality itself and in others cities, sometimes out of the state. However, to minimize this limitation, these students were invited to participate by e-mail, responding online to the instrument through an electronic form.

## Conclusion

The intensity of common symptoms of depression ranged from moderate to severe in 23.6% of undergraduate health students. The courses with the highest prevalence of common symptoms of moderate to severe depression were Speech Therapy (47.6%) and Nursing (34.2%).

Regarding the variables associated with common symptoms of depression, those who did not perform physical activities, had no leisure time and attended speech therapy and nursing courses showed a higher prevalence of symptoms. In addition to these, we identified an association with females, using bus as a means of transport, having some medical diagnosis, making use of medication and being obese.

These variables associated with common symptoms of depression may be related to negative thoughts; distortions and perception patterns of reality; a stressful routine, which involves various academic and extra-classroom activities; and a high course load. In this sense, both individual and collective efforts are important, that is, both by the student, who should better organize and manage time, and by the university itself, which should review its curricula, activities, as well as its role in the development of strategies to promote well-being based on policies and institutional services to protect the physical and mental health of these students. Actions to promote physical and leisure activities, in addition to identifying weaknesses in the courses, can lead to better academic achievement and the personal and professional development of the student.
